# Periodontal Pathogens and Their Links to Neuroinflammation and Neurodegeneration

**DOI:** 10.3390/microorganisms11071832

**Published:** 2023-07-18

**Authors:** David Visentin, Ivana Gobin, Željka Maglica

**Affiliations:** 1 Department of Biotechnology, University of Rijeka, 51000 Rijeka, Croatia; david.visentin@uniri.hr; 2 Department of Microbiology and Parasitology, Faculty of Medicine, University of Rijeka, 51000 Rijeka, Croatia; ivana.gobin@uniri.hr

**Keywords:** periodontal pathogens, neuroinflammation, neurodegeneration

## Abstract

Pathogens that play a role in the development and progression of periodontitis have gained significant attention due to their implications in the onset of various systemic diseases. Periodontitis is characterized as an inflammatory disease of the gingival tissue that is mainly caused by bacterial pathogens. Among them, *Porphyromonas gingivalis*, *Treponema denticola*, *Fusobacterium nucleatum*, *Aggregatibacter actinomycetemcomitans*, and *Tannerella forsythia* are regarded as the main periodontal pathogens. These pathogens elicit the release of cytokines, which in combination with their virulence factors induce chronic systemic inflammation and subsequently impact neural function while also altering the permeability of the blood–brain barrier. The primary objective of this review is to summarize the existing information regarding periodontal pathogens, their virulence factors, and their potential association with neuroinflammation and neurodegenerative diseases. We systematically reviewed longitudinal studies that investigated the association between periodontal disease and the onset of neurodegenerative disorders. Out of the 24 studies examined, 20 showed some degree of positive correlation between periodontal disease and neurodegenerative disorders, with studies focusing on cognitive function demonstrating the most robust effects. Therefore, periodontal pathogens might represent an exciting new approach to develop novel preventive treatments for neurodegenerative diseases.

## 1. Introduction

The oral cavity comprises a diverse array of microbiological environments, owing to the local dynamic mechano-chemical conditions. These fluc.tuations in the environment give rise to multiple ecological niches that support the growth of a wide range of oral pathogens. To date, over 700 species of microorganisms have been identified in the oral cavity, out of which 500 are bacterial species [1]. These species are mostly comprised of several phyla including Actinobacteria, Proteobacteria, Firmicutes, Bacteroidetes, Tenericutes, Euryarchaeota, Chlamydiae, and Spirochaetes [2].

One location within the oral cavity that is particularly conducive to bacterial growth is the gingiva, the tissue surrounding the base of the teeth [3]. The gingiva, owing to its proximity to the teeth and its rich nutrient supply, can be susceptible to bacterial overpopulation in the event of poor oral hygiene. Such bacterial proliferation in the gingival sulcus can lead to the development of gingivitis, a common oral disease [4].

### Gingivitis and Its Progression to Periodontitis

Gingivitis is a common form of gum disease that can cause irritation and inflammation of the gingival tissue. The onset of gingivitis is multifactorial and can be caused by the formation of plaque, as well as changes in nutrition, hormone levels, and drug abuse [4]. Specific species are implicated in the initiation and progression of gingivitis and include species of *Streptococci*, *Fusobacteria*, *Actinomyces*, *Veillonella*, *Leptotrichia*, *Prevotella*, *Treponema*, with *Bacteroides*, *Capnocytophaga*, and *Eikenella* also possibly playing an important role [5].

The disease has four stages of progression, with each stage increasing in severity and microbial diversity. As characterized by Page and Schroeder, the stages of gingivitis are “initial lesion” “early lesion”, “established lesion”, and “advanced lesion” [4]. The initial lesion is an acute inflammation that is followed by increased gingival fluid flow and recruitment of neutrophils. The early lesion is characterized by lymphoid cell infiltration (predominantly of T cells). This is also the stage where clinical signs of gingivitis, such as redness and bleeding, start appearing. Progressive worsening of the clinical condition leads to the formation of established lesions called gingival pockets, in which B lymphocytes and plasma cells predominate. This phase can either persist indefinitely, revert to an earlier stage, or progress to an advanced lesion, which is a transition phase to another disease called periodontitis [4,6]. Currently, 20–50% of the global population is affected by some form of periodontal disease, with 10.8% of them displaying symptoms of severe chronic periodontitis [7,8].

It is not well understood how changes in the oral microbiota contribute to the progression of gingivitis, but evidence suggests that some hosts are more susceptible to the disease. These individuals show a tendency to more rapid clinical deterioration due to the differences in their baseline healthy oral microbiota. Such hosts had an increase in certain bacteria from the genera *Selenomonas*, *Lachnospiraceae*, *Peptococci*, *Bacteroidaceae*, *Peptostreptococci*, *Oribacteria,* and *Veillonellaceae,* as well as a decrease in *Abiotrophia* [9].

The progression of the disease to periodontitis is affected by multiple local and systemic etiological factors. For that reason, the presence of microbial biofilm is not always sufficient for the pathogenesis of periodontitis. Instead, the disease arises from an imbalance between the host and the microbial biofilm. Further understanding of the imbalance that presets the disease is challenging, given the large variation in the oral microbiota, dental plaque, and the host genetic and immune system profiles [10]. Studies suggest that during gingivitis there is an increase in both the richness and diversity of the subgingival microbiota, and while microbiome diversity in periodontitis remains increased, some species become dominant, reducing the overall diversity when compared to gingivitis [11].

The untreated inflammation caused by periodontal pathogens spreads to the deeper tissue of the teeth and causes tissue attachment loss, followed by the migration and withdrawal of the junctional epithelium. These changes result in the formation of periodontal ‘pockets’, which are considered a hallmark of periodontitis [4]. Prolonged inflammation of the deeper tissues causes an alteration in bone homeostasis and can result in the complete disintegration of the connecting tissue followed by tooth loss [4,10].

## 2. Periodontal Pathogens, Virulence Factors and Host Response

The prevalence of certain bacterial species and their virulence factors have been shown to be directly related to susceptibility, installation, and progression of periodontitis. Bacterial species present in the subgingival biofilms were sorted by color into six bacterial complexes [12]. The discussion of which species are particularly virulent and initiate the disease is still ongoing, but a specific group of bacteria characterized as the red complex is considered to encompass the most important pathogens in adult periodontal disease. This complex comprises of *Porphyromonas gingivalis*, *Treponema denticola*, and *Tannerella forsythia* [10,13]. Additionally, some bacteria from outside this complex, such as *Fusobacterium nucleatum*, *Aggregatibacter actinomycetemcomitans*, *Prevotella* species, *Eikenella corrodens*, *Peptostreptococcus micros*, and *Campylobacter rectus*, have been shown to play a possible role in disease pathogenesis [10,13,14]. As of now, *P. gingivalis*, *T. denticola*, *T. forsythia*, *A. actinomycetemcomitans*, and *F. nucleatum* are regarded as the principal periodontal pathogens [10,14].

### 2.1. Porphyromonas gingivalis

*P. gingivalis* is a Gram-negative anaerobic bacteria that has been identified as a keystone pathogen in the development of periodontitis. Its prevalence is directly associated with the severity of the disease, leading to extensive research on the bacteria’s role in oral health as well as its association with other diseases [15,16].

*P. gingivalis* has several specialized properties and virulence factors that contribute to its unique impact on the host [15]. One such property is the shedding of outer membrane vesicles (OMVs), which contain the bacterium’s virulence factors, notably gingipains and lipopolysaccharide (LPS) [17,18]. Gingipains are cell surface trypsin-like cysteine proteinases that are crucial for the pathogenicity of *P. gingivalis*, consisting of lysine-specific (Kgp) and arginine-specific (RgpA and RgpB) proteinases [19]. These enzymes are potent pro-inflammatory factors that trigger the release of the IL-1β and TNF-α via NLR family pyrin domain-containing protein 3 (NLRP3) while also regulating the host’s response by cleaving IL-1β and TNF-α and degrading macrophage CD14 which is crucial for LPS-induced activation of the leukocytes [19,20].

In addition to their pro-inflammatory effects, gingipains also inhibit the phosphoinositide 3-kinase (PI3K)/protein kinase B (AKT) pathway, facilitate bacterial coaggregation with other oral bacteria. They also dysregulate the complement cascade by cleavage of C3 into C3a and C3b followed by subsequent degradation of C3a and C3b, preventing the formation of C5 convertase [20]. Gingipains play a particularly important role in periodontitis by enhancing vascular permeability through the activation of the kallikrein/kinin pathway. Kgp is primarily involved in gingival bleeding, while RgpA and RgpB activate coagulation factors and degrade fibrinogen/fibrin [19,20]. LPS also plays a role in promoting tissue destruction through its pro-inflammatory response upon binding with TLR-2 and TLR-4 [20]. This causes a downstream activation of signaling pathways such as activator protein 1 (AP-1), nuclear factor kappa B (NF-κB), mitogen-activated protein kinase (MAPK), c-Jun N-terminal kinases (JNK), and PI3K/Akt, which, in turn, promotes the release of pro-inflammatory factors such as IL-1β, IL-6, and IL-8 and up-regulation of Th17 cell differentiation with LPS derived from a more virulent strain, having a higher impact [15,20,21,22,23]. *P. gingivalis* also causes SerB phosphatase-dependent alveolar bone loss, partially through stimulation of macrophages [24,25]. Some other important factors of *P. gingivalis* are peptidylarginine deiminase (PADs), heat-shock proteins (GroEL), and an unidentified soluble heat-stable component that was shown to promote lymphocyte apoptosis in peripheral blood mononuclear cells [15,26]. Besides its virulence factors, *P. gingivalis* is also able to directly cause a pro-inflammatory response through a fimbria-dependent infection of dendritic cells. This causes dendritic cells to undergo maturation, causing a secretion of IL-1β, IL-6, TNF-α, IL-10, IL-12, IFN-γ, and dendritic cell-induced proliferation of autologous CD4(+) T cells [27].

*P. gingivalis* is also able to evade the immune system through a number of mechanisms, including the production of immunoglobulin-binding proteins and the suppression and evasion of phagocytosis [28,29]. To further avoid the host immune system, the bacteria can replicate inside autophagosome vacuoles in human coronary artery endothelial cells [30]. 

It is difficult to determine the exact impact that *P. gingivalis* has on the host due to its genetic variability between strains. To date, genomic sequences of 94 strains have been determined, with a high degree of variation in their pathogenicity and functional properties [31]. Most of the observed differences in virulence between strains result from differences in the presence of the capsule and its composition, hemagglutinins, fimbriae composition and abundance, and gingipain production [32,33]. Notably, certain *P. gingivalis* strains are predominantly found in healthy individuals, while others are highly associated with the development and severity of periodontitis. In particular, the CP3 strain of *P. gingivalis*, isolated from chronic periodontitis patients, has been found to exhibit six-fold higher invasion capabilities than the H3 strain, which in contrast was isolated from healthy individuals [32]. The main strains that are currently used in research are the less virulent ATCC 33277 and FDC 381, and the more virulent W50 and W83 Table 1 [34].

### 2.2. Tannerella forsythia

*T. forsythia* (formerly referred to as *Bacteroides forsythia*) is a Gram-negative anaerobic pathogen and a principal agent of periodontitis. However, due to difficulties in cultivating this organism, research on *T. forsythia* has been limited until recently [41]. In vitro studies have demonstrated that *T. forsythia* can cause alveolar bone reabsorption and cutaneous abscesses in mice [41]. 

Unlike some other pathogens, *T. forsythia* utilizes proteins as a source of energy and is unable to breakdown sugars [42]. To break the peptides, the bacteria employ trypsin-like proteases and cysteine-like proteases (PrtH) [41]. While trypsin-like proteases contribute little to the disease, PrtH is the major primary virulence factor of *T. forsythia* studied to date. PrtH was initially described as Forsythia detaching factor because of its role in cell separation and destruction of the subgingival epithelium [41,43]. Additionally, PrtH has been shown to increase the mitochondrial oxidative membrane potential in vitro, thereby stimulating the production of IL-8 [44]. The leucine-rich repeat cell-surface and secreted protein, BspA, has been shown to be required for attachment to and invasion of epithelial cells and can induce chemokine expression via binding with TLR2 [45]. It was also observed that BspA can alter the progression of atherosclerotic lesions in ApoE(−/−) mice [46]. *T. forsythia* also produces an electrophilic compound, methylglyoxal, which can generate inflammatory adducts by covalent modification of amino acid side chains to create advanced glycation end-products (AGEs). The resulting AGEs increase pro inflammatory and pro-osteoclastogenic activity [47]. Other interesting virulence factors of *T. forsythia* include GroEL and karilysin. GroEL is a heat shock protein that synergizes with cytokine IL-17, stimulating inflammatory bone resorption [48]. Karilysin is a matrix metalloprotease-like enzyme that can regulate TNF-α concentration in serum by promoting functional TNF-α shedding from macrophage surfaces through proteolytic cleavage, increasing the total TNF-α concentration [49,50].

Besides proteolytic activity, *T. forsythia* possesses immune evasion capabilities, in part thanks to its S-layer, which is a unique surface layer formed of the glycosylated proteins TfsA-GP and TfsB-GP [51]. It impacts the adhesion and invasion of cells, bacterial coaggregation and attenuates the expression of pro-inflammatory cytokine by evading immune recognition [52,53,54]. Furthermore, the metalloproteinase karilysin inhibits all pathways of the complement system and is involved in the bacteria’s serum resistance [55]. Interestingly, *T. forsythia* also exhibits cytopathic activity arresting cells at G2. However, the underlying factor remains to be identified [43].

The impact of strain variances on the behavior of *T. forsythia* remains poorly understood. Even so, some differences have been noted between the primary *T. forsythia* strains, which mostly involve changes in biofilm formation and the bacterium’s specificity and binding to *P. gingivalis* [56,57]. Nonetheless, some strain-dependent changes in host immunological responses have also been observed. For instance, the *T. forsythia* UB20 strain was shown to induce higher secretion of chemokine protein CXCL10 than the ATCC 43037 strain, possibly due to the difference in LPS [58].

### 2.3. Treponema denticola

*T. denticola* is Gram-negative anaerobic bacteria from the Spirochetes family with high motility and proteolytic ability. It triggers an immunological response mainly via TLR2, predominantly through its periplasmic flagellum [59]. The outer membrane of the bacteria, also known as the outer sheath, contains two main pro-inflammatory factors: major sheath protein (MSP) and lipooligosaccharide (LOS). MSP was shown to have a cytotoxic pore-forming activity and induce a pro-inflammatory response (TNF-α, IL-1β, IL-6, and MMP-9) while also being able to disrupt the intracellular regulatory pathways of infected host cells [60,61,62]. LOS, on the other hand, can bind to extracellular matrix proteins, mucosal cells, and oral bacteria [63]. It can also affect binding to other bacteria, thereby increasing their pro-inflammatory potential [63]. Macrophage exposure to LOS and MSP induces tolerance to further stimulation with enterobacterial LPS [62]. Another major virulence factor of *T. denticola* is the prolyl phenylalanine-specific peptidase dentilisin, which is a surface-expressed protease complex comprised of three lipoproteins. It degrades host cell proteins, stimulates tissue destruction in a TLR2/MyD88/Sp1-dependent fashion, and activates polymorphonuclear neutrophils via the complement C3 pathway [64,65]. Furthermore, dentilisin contributes to the motility of the bacterium by facilitating crawling-dependent surface spreading [66].

Interestingly, *T. denticola* has a pronounced ability to evade and suppress immunological responses even when compared within its own genus [67]. *T. denticola* fails to induce RANTES, IL-8, and human beta-defensin-2 messenger RNA response in human gingival epithelial cells [68]. Instead, it reduces protein expression of human beta-defensins (HBDs, reduced by 40%), TNF-α and IL-8. This is in part due to the inhibition of the TLR2 axis by unknown heat-labile inhibitor(s) and dentilisin-dependent cleavage of TNF-α which subsequently also causes a downregulation of IL-8, HBD-2, and HBD-3, significantly dampening the host’s immunological response [68,69,70]. Dentilisin is also responsible for the direct degradation of IL-8 and some other immunological factors, most importantly IgG [71,72]. Despite this, the bacteria still induces minimal macrophage-mediated inflammation [72].

Recent studies have identified significant strain variation in the biofilm formation capabilities of *T. denticola*. This could be partially dependent on the bacterium’s mobility, which was shown to differ greatly between strains [73].

### 2.4. Fusobacterium nucleatum

*F. nucleatum* is a Gram-negative, anaerobic oral commensal, that plays a crucial role in the formation of dental plaque through its ability to strongly coaggregate with other bacteria [74]. Importantly, *F. nucleatum* and its subspecies have been found to be among the most abundant bacteria in cases of periodontitis [11]. While the bacteria has primarily been implicated in this oral disease, recent evidence has also linked it to a variety of systemic illnesses, including adverse pregnancy outcomes, rheumatoid arthritis, organ abscesses, and gastrointestinal disorders, most importantly colorectal cancer [74]. Experimental data also suggests that *F. nucleatum* is capable of causing systemic inflammation, promoting cancer metastasis, and increasing drug resistance [74,75,76].

In the context of periodontitis, *F. nucleatum* has been shown to increase the expression of cytokine and chemokine genes, such as those encoding for TNF-α, IL-8, IL-6, CCL2, and CXCL1 while also being capable of significantly inhibiting cell proliferation of gingival-derived mesenchymal stem cells and gingival fibroblasts [74,77]. Additionally, in gingival fibroblasts, the bacteria induces ROS generation and apoptosis through activation of the AKT/MAPK and NF-κB signaling pathways [78]. Its LPS is known to cause inflammatory degradation of host tissue and bone resorption, mainly by stimulating MMP-9 secretion from macrophages through the TLR4/MyD88 axis [79].

The primary virulence factor of *F. nucleatum* is FadA, an adhesin protein that is required for the invasion and adhesion of the bacterium to host cells, while also increasing the permeability of endothelial cells to other bacteria [80]. The bacteria also secrete fusolisin, a serine protease that degrades extracellular matrix proteins and IgA [81]. As mentioned previously, an important property of the bacteria is assistance in bacterial coaggregation, in which the outer membrane proteins RadD and Fap2 function as adhesins, binding to a variety of bacteria while also inducing lymphocyte apoptosis [74,80]. Furthermore, *F. nucleatum* has been shown to have the capability to modulate the host cell transcriptome and epigenome. On exposure to the bacteria, human colonic epithelial cells display changes in gene expression associated with p53 degradation-induced proliferation and release of ROS, while in human carotid artery endothelial cells, exposure causes overexpression of *EFNA1* and *LIF*, two genes connected to colorectal cancer and down regulation of multiple histone modifications related genes [82]. It was also shown that *F. nucleatum* can modulate the osteogenic and dentinogenic potential of human stem cells from the Apical Papilla (tissue located at the base of the developing dental root) in vitro [83].

*F. nucleatum* is a heterogenous species, exhibiting considerable genetic variability, with the number of *F. nucleatum*-specific genes varying in count up to ten-fold between strains [80,84]. For that reason, *F. nucleatum* is currently subdivided into four subspecies: *animalis*, *nucleatum*, *polymorphum*, and *vincentii* (which includes *fusiforme*) [80]. These subspecies exhibit varying production of virulence factors, as well as differing in the ability to form biofilms and attach to and invade cells due to changes in their surface-expressed glycans and proteins [80,84,85]. For instance, *F. nucleatum* ssp. *polymorphum* is the only subspecies that failed to form a single-subspecies biofilm in vitro, likely due to its less conserved adhesion proteins CmpA and Fap2 [85].

### 2.5. Aggregatibacter actinomycetemcomitans

Another significant periodontal pathogen is *A. actinomycetemcomitans*, a Gram-negative non-motile anaerobe. It is mainly known for its early colonization of the gingiva and its high association with the severity and progression of periodontitis [86]. The bacteria’s LPS stimulates collagen phagocytosis, alongside IL-8 and IL-6 production from gingival fibroblasts and an increase in IL-12, IFN-γ, TNF-α, IL-1β, and IL-6 production in dendritic cells [87,88,89]. Its LPS has also been shown to be a potent stimulant of ROS in neutrophils [90]. In addition to LPS, *A. actinomycetemcomitans* produces several other virulence factors, including leukotoxins and cytolethal distending toxins (CDTs). Leukotoxins, which are localized on the outer membrane of the bacterium and are secreted into the serum, bind to the lymphocyte function-associated antigen 1 (LFA-1) CD18 subunit and cause a suppressed immune response, allowing for the progression of periodontitis [91]. The effects of leukotoxins on different leukocyte populations vary with neutrophils, monocytes, and other cells being affected differently [91]. There is a great difference in genetic diversity of *A. actinomycetemcomitans*, with substantially different virulence properties, partially owing to a difference in leukotoxin production [86]. CDTs are bacterial protein exotoxins that are expressed by several Gram-negative species. They are genotoxins that cause DNA damage, cell cycle arrest, and eventually apoptosis and are highly toxic to immune cells, including T cells, B cells, and mononuclear cells [92]. CDTs were also shown to stimulate the production of pro-inflammatory factors in peripheral blood mononuclear cells and osteolytic cytokines in periodontal connective tissue cells, such as RANKL, which leads to bone resorption in periodontitis [93]. 

Currently there are seven different serotypes of *A. actinomycetemcomitans* (a–g), which are differentiated based on their surface antigens, with certain serotypes exhibiting higher pathogenicity than others [94]. This variation in pathogenicity is believed to be primarily due to changes and deletions within the promoter region of a gene that regulates leukotoxin expression [94,95]. Notably, some highly pathogenic strains that belong to the group of serotype b strains (designated as JP2 clone) exhibit a 530 base pair deletion in the promotor region, resulting in a 10 to 20-fold increased production of leukotoxin in comparison with other strains [94,96].

A summary of the main virulence mechanisms of each bacterium can be seen in Table 2.

### 2.6. Synergistic Behavior

Periodontal bacteria rarely exist in isolation and require the presence of other bacterial species for optimal growth and function [102]. Understanding the underlying synergistic interactions among periodontal bacteria is crucial for comprehending oral microbial pathogenesis and its implications for neurodegenerative disorders. Co-aggregation, modulation of virulence factors, and the impact of nutrient availability, all play an important role in the formation of the disease’s phenotype [103].

Among the main periodontal pathogens, *P. gingivalis* has been extensively studied for its interactions with other bacteria, demonstrating synergistic interactions with all of them. For instance, it directly co-aggregates with *F. nucleatum* and *T. denticola* forming complex biofilms [104]. Gingipains that are released from *P. gingivalis* support the growth of various microorganisms by increasing nutrient availability while also protecting the bacteria from the immunological response of the host [14]. Additionally, gingipains enhance the attachment and invasion of *T. forsythia* to epithelial cells [105]. *P. gingivalis* increases the free glycine in the surrounding media, which was shown to support *T. denticola* growth [106,107]. The bacteria also enhances biofilm formation by *F. nucleatum* by releasing diffusible signaling molecules [108].

On the other hand, *F. nucleatum* was shown to enhance the growth of *P. gingivalis* in the presence of oxygen, which suggests that *F. nucleatum* is capable of lowering local redox potential, thus benefiting the growth of more oxygen-sensitive anaerobic organisms [109]. Mixed infection with *F. nucleatum* was also shown to strengthen the invasion capacity of *P. gingivalis* in oral epithelial cells [110]. Co-culture with *T. denticola* results in an increase in *P. gingivalis* biomass, possibly through succinate released from *T. denticola*, which has been shown to alleviate the heme requirement of *P. gingivalis* when grown in a heme-limited environment [107]. Sonicated extracts of *T. forsythia* stimulate growth of *P. gingivalis* in nutrition-decreased medium, possibly due to a protein acting as a growth-promoting factor [111]. While the extent of interactions between *A. actinomycetemcomitans* and *P. gingivalis* are not yet fully studied, a synergistic effect on pathogenicity has been reported [112]. 

**Figure 1:**
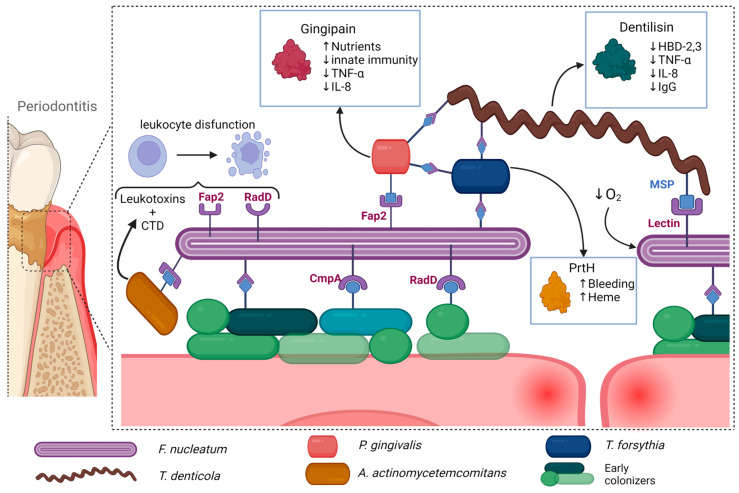
Periodontal pathogens exhibit a range of synergistic interactions that facilitate further bacterial growth and proinflammatory pathways. For instance, certain species such as *F. nucleatum* and *T. denticola* act as scaffolding bacteria, binding to other periodontal pathogens and allowing for the formation of complex biofilms. *T. denticola* also exhibits a strong immunoregulatory effect, reducing the efficiency of the host’s immune response, while *F. nucleatum* decreases local oxygen concentration, further favoring the growth of periodontal pathogens. Other species such as *P. gingivalis* produce gingipains that increase nutrient availability, while also reducing the effectiveness of the host’s immunological response. *A. actinomycetemcomitans* also exhibits a strong immunoregulatory effect through leukotoxin and CTD, which inhibit the function of leukocytes. *T. forsythia* further degrades epithelial cells, increasing nutrient and heme availability. Taken together, these interactions contribute to the progression of periodontitis and its potential systemic consequences. Created with BioRender.com, accessed on 3 July 2023.

Other periodontal pathogens, although less studied, also exhibit various synergistic interactions. *T. denticola* MSP binds to *F. nucleatum* and facilitates bacterial coaggregation, while *F. nucleatum* promotes bacterial growth [104,113]. It was also noted that *F. nucleatum* and *T. forsythia* develop synergistic interactions when co-cultured [114]. Hydrolyzed β-glucan released by *T. forsythia* β-glucanase supports *F. nucleatum* growth, while *T. forsythia* can utilize muropeptides derived from *F. nucleatum* [115,116]. Co-culture of *T. forsythia* along with other periodontal pathogens enhanced the formation of abscesses in rabbits and mice, while also inducing a synergistic effect on alveolar bone loss [117]. Some of the more prominent interactions can be seen in Figure 1.

### 2.7. Mechanisms of Systemic Inflammation and Its Correlation to Periodontitis

The infection of periodontal pathogens and subsequent release of cytokines have been shown to induce low-grade, chronic systemic inflammation in patients. Elevations of pro-inflammatory mediators, such as IL-1, IL-6, C-reactive protein (CRP), and fibrinogen, as well as increased neutrophil count in the blood, have been observed in individuals with periodontal disease [118,119,120]. Additionally, successful periodontal treatment has been shown to reverse systemic inflammatory marker levels in the blood, suggesting a causal relationship between periodontal disease and systemic inflammation [119].

**Figure 2:**
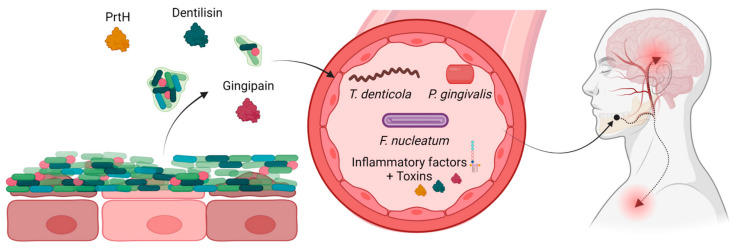
Periodontitis produces bacterial toxins and proinflammatory factors, which alongside detached bacteria can enter the blood stream, disseminating and possibly causing neural inflammation. Created with BioRender.com, accessed on 12 May 2023.

The underlying mechanism of this phenomenon is believed to be the increase of bacterial load, which leads to a weakening of the barrier separating the biofilm from the blood stream, allowing for the dissemination of pro-inflammatory factors from the periodontal bacteria and infected tissue [121,122]. This process may also permit bacterial products, such as lipopolysaccharides, outer membrane vesicles, and proteases, to reach the circulation, resulting in bacteremia as shown in Figure 2 [121]. Furthermore, certain periodontal bacteria possess the ability to disseminate to other parts of the body, likely via the bloodstream, although recent evidence suggests that other bacteria, like *T. denticola*, may utilize the lymphatic system instead [16,123].

This capability to disseminate further enhances systemic inflammation and may help explain the link between periodontitis and a range of systemic diseases. Such diseases include cardiovascular disease, rheumatoid arthritis, respiratory diseases, chronic kidney disease, metabolic disease (such as type II diabetes, and metabolic syndrome-related obesity), renal disease, non-alcoholic fatty liver disease, gut microbiome-related disorder (such as adverse pregnancy outcomes, celiac disease, inflammatory bowel disease, and irritable bowel syndrome), impairment of cognitive function, asthma, allergy, wound closure, cancers, aneurysms, stroke, microvascular defects, age-related disorders, regenerative, and stem cell dysfunction [122,124]. In recent years, exploring the linkages between periodontal pathogens and chronic diseases has been a focal point of extensive research endeavors. In addition to the well-documented impact of the inflammatory response, several theories have been proposed to clarify the underlying mechanisms driving this intricate relationship, including the release of specific factors that facilitate disease progression, cellular infections leading to alterations in protein expression that favor pathogenesis, interactions with receptors, and immunomodulatory effects [125].

The relationship between inflammation and altered brain function can be seen through elevated serum levels of pro-inflammatory cytokines, such as IL-1, IL-6, and TNF-α, in patients with depression [126]. Tricyclic antidepressants can inhibit cytokine release in immune cells, while therapies involving IFN- α, and IL-2 can result in depression-like symptoms [127,128,129].

It is noteworthy that periodontal pathogens have consistently been detected in the interdental biofilm of periodontally healthy subjects, challenging the assumption that these bacteria are exclusive to those with clinical inflammation [130,131,132]. Observed periodontal bacteria, specifically from the red complex, have been positively linked to an increased risk of caries through the disruption of the local microbial flora [132]. Moreover, a reduction in the levels of red and orange Socransky complexes bacteria in subjects that display no signs of gingivitis has been associated with a significant decrease in interdental inflammation [131]. This suggests that the pathogenic processes of periodontal bacteria can be triggered in healthy subjects and cause a state of para-inflammation, preceding the manifestation of clinical symptoms by several years or decades.

## 3. Neurodegenerative and Periodontal Disease

Neurodegenerative diseases are a diverse array of conditions that affect the central and peripheral nervous system and includes Alzheimer’s disease (AD), Parkinson’s disease (PD), multiple sclerosis (MS), amyloid lateral sclerosis (ALS), Huntington’s disease, and various other neurodegenerative disorders [133]. It is characterized by impaired and eventual loss of neuronal function, often culminating in cognitive decline, intellectual deterioration, and dementia. While neurodegenerative diseases all have different pathways of onset and progression, they all share similar mechanisms which can in the majority be grouped in one of four categories: 1—Genetic Predisposition and Environmental Factors; 2—Protein Dysregulation, Aggregation, and Neurodegeneration; 3—Neuroinflammation and Inflammatory Factors; 4—Blood–brain barrier Dysfunction and Peripheral Immune Activation [134]. Over the past decade, neuroinflammation has been getting more attention due to discoveries of its vast implications for all neurodegenerative diseases. It is characterized by the activation of glial cells and the release of inflammatory factors [134]. Probably the best known neuroinflammatory disorder is multiple sclerosis which is considered a T-cell mediated autoimmune disease and is characterized by continuous neuroinflammation and demyelination [135]. In Alzheimer’s disease, increased levels of pro-inflammatory cytokines, such as IL-1β, IL-6, and TNF-α, have been observed in the brains of affected individuals [136]. Moreover, studies have shown that these inflammatory factors can contribute to the production of amyloid plaques and tau hyperphosphorylation, further exacerbating neuronal dysfunction [136]. In Parkinson’s disease, microglial activation and subsequent release of inflammatory mediators, including TNF-α, IL-1β, and prostaglandins, contribute to the neuroinflammatory response and progressive neurodegeneration [137].

Emerging evidence suggests that blood–brain barrier (BBB) dysfunction and peripheral immune activation play crucial roles in the initiation and progression of neurodegenerative diseases [138]. Studies utilizing advanced imaging techniques and biomarker analysis have revealed BBB disruption in individuals with AD and MS, characterized by increased permeability, and altered expression of tight junction proteins [139,140]. Furthermore, peripheral immune cells, including monocytes and T cells, can infiltrate the CNS in response to chronic inflammation, releasing inflammatory factors that contribute to neuroinflammation and neurodegeneration [141].

### 3.1. Periodontitis and Neurodegenerative Disorders

In recent years, there has been growing interest in the potential link between periodontitis and the onset and progression of neurodegenerative disorders. Variations in testing methods and demographics make it challenging to establish a clear causal connection. However, evidence suggests that pro-inflammatory mediators produced in response to periodontal pathogens can alter the permeability of the blood–brain barrier and pass through it, which leads to neuroinflammation and synaptic impairment [22,142,143,144]. For this reason, multiple studies have tried to observe the possible interaction periodontitis would have with neurodegenerative disorders as shown in Table 3. Criteria and the process of selecting studies that were then included in Table 3 can be seen in Appendix A.

The currently available and acquired longitudinal studies are as follows: 1 study that focuses on mild memory impairment, 5 studies for mild cognitive impairment, 14 studies for Alzheimer’s and other dementias, and 4 studies that focus on Parkinson’s disease. Out of the 24 studies, 20 found some form of positive correlation between periodontal disease and neurodegenerative disorders, with the highest consistent effect being observed in studies that focused on cognitive function. 

Additional studies have revealed a higher number of positive IgG antibody tests against *A. actinomycetemcomitans*, *P. gingivalis*, and *T. forsythia* in subjects with AD [169]. Furthermore, some periodontal pathogens, such as *P. gingivalis*, *T. denticola*, and *F. nucleatum*, were shown to be able to directly enter the central nervous system and trigger neuroinflammation [16,123,170,171]. Postmortem studies showed that 8 out of 10 AD patients had *P. gingivalis* DNA present in their cerebrospinal fluid, with the correlation between gingipains load in the brain and AD diagnosis and pathology [16]. Another study used brain imaging to observe the impact of periodontal treatment on imaging markers of AD and found a moderate to strong protective effect [172].

Animal research was conducted to further investigate this correlation, using models of periodontitis in rats and mice. When subjected to these models, animals exhibited cognitive impairment and a significant reduction in a number of neurons, as well as an increase of pro-inflammatory factors (IL-1β and TNF-α), in the hippocampus [173,174,175]. This was accompanied by an increase in Tau phosphorylation, as well as expression of the glial fibrillary acidic protein, IL-6, cyclooxygenase-2, iNOS, AβPP, and β-secretase 1 and a decrease in A disintegrin and metalloproteinase domain-containing protein 10 expression [173,174]. Additionally, oral administration of *P. gingivalis* alone also leads to the activation of microglial cells and overexpression of pro-inflammatory cytokine (IL-6, TNF-α, and IL-1β), followed by neuronal death in the cortex, hippocampus, and substantia nigra [16,176,177]. Infected mice exhibit pathological features similar to those of Alzheimer’s disease, including elevated expression of the APP and BACE1 genes and an increase in tau cleavage that is dependent on gingipain. This results in elevated production of the Aβ1-42 peptide [16,177].

In one study, mice treated with *P. gingivalis* displayed depression-like behavior possibly caused by LPS-TLR4-dependent downregulation of p75NTR in astrocites [178]. Administration of LPS alone in the gingival tissue of mice was also shown to stimulate glial cell production of inflammatory cytokines which then afflicted the cortex and hippocampus resulting in significantly impaired spatial learning and memory [22]. *P. gingivalis* was also shown to regulate the neuronal cell cycle by upregulating *E2F1* and downregulating *CDK11*, and *iNOS* gene expression [179].

The idea that oral bacteria can affect neuronal health is still relatively new and, therefore, not much is known about the specific impact of other periodontal pathogens. However, similar findings have been made for *T. denticola*, which was shown to be capable of entering the brain and directly impacting nerve cells. This resulted in the accumulation of intra and extracellular Aβ and neuronal apoptosis [180,181].

### 3.2. Bidirectional Interactions, Causation, and Critical Overview

Neurodegenerative disorders are amongst the most complex and enigmatic of diseases and are influenced by multiple systemic, genetic, and environmental factors which all modify the onset and progression of the disease. The relationship between periodontitis and neurodegenerative disorders is similarly complex and not yet fully understood, with no direct causal relationship established to date. 

Studies investigating the relationship between periodontitis and dementia have yielded mixed results as shown in Table 3. Regrettably, there were no longitudinal studies that fit the criteria regarding MS, ALS, and Huntington’s disease. Particularly in the context of MS, investigating the potential influence of periodontal disease on its onset and progression holds considerable interest, given the established association with neuroinflammation. However, an investigation conducted in 2015, employing a case–control design with 756 affected patients, failed to establish a significant correlation between periodontitis and MS after adjusting for confounding factors such as smoking [182]. Furthermore, a comprehensive review of 17 studies in 2021 that explored the relationship between MS and oral health yielded no evidence of an association [183]. It is possible that the inflammatory processes that occur in MS may supersede the modest influence exerted by the introduction of further inflammation through periodontitis. To gain deeper insights into this interplay, further investigation is required.

While periodontitis and neurodegenerative diseases are etiologically different, they share multiple common risk factors such as hypertension and diabetes. Another such unaccounted for confounding variable may be responsible for the observed correlation. As Thomson et al. pointed out people with better childhood cognitive function have better oral health which makes them more likely to have better cognition in old age [151]; therefore, it is difficult to ascertain if periodontitis is a risk factor for neurodegeneration or if both conditions are coexisting due to shared risk factors. If it is established that periodontitis does directly impact neurodegeneration, it is unlikely to be a clear-cut relationship. It is possible that periodontal bacteria only impact neurodegeneration after disseminating to the brain, which would make the pathology invisible as long as the blood–brain barrier remains intact.

The significant variation in the behavior of different bacterial strains and their local interactions with other species further complicates the issue. While some strains may not impact neurodegeneration, others could have a significant effect. This could also vary depending on local synergistic interactions the pathogenic strain forms with the surrounding species, which could again be strain dependent. However, current testing methods do not account for strain type, leading to inconsistency. Nonetheless, further research is required to fully comprehend the relationship between periodontitis and neurodegeneration.

## 4. Conclusions

Periodontal disease is a preventable and prevalent condition that could have an impact on neurodegenerative function through the induction of chronic, low-grade systemic inflammation. The resulting pro-inflammatory factors can increase the permeability of the blood–brain barrier, allowing for the infiltration of pathogens or pro-inflammatory agents into the brain, leading to neuroinflammation and synaptic impairment, which could contribute to neurodegenerative disorders such as Alzheimer’s disease. However, it should be emphasized that although there is a significant amount of clinical evidence indicating a potential association between periodontitis and neurodegenerative disorders, proving causation, and determining the strength of this association is challenging. To better understand this relationship, further research is required.

## Figures and Tables

**Table 1:** Comparison and summary of the main differences between widely used strains of *P. gingivalis*.

Strain	Genome Size (bp)	Capsule	Fimbriae/Type	Mono-Species Biofilm Formation	Gingipain Activity	Virulence Potential	Citation
ATCC 33277	2,354,886	none	Abundant/Type I	Moderate	High	Moderate	[15,32,33,34,35,36,37,38]
FDC 381	2,378,872	none	Abundant/Type I	Moderate	Medium	Moderate	[15,33,34,36,37,38,39]
W83	2,343,476	K1 encapsulated	Poor/Type IV	None	High	High	[15,32,34,35,36,37,38]
W50	2,345,841	K1 encapsulated	Poor/Type IV	None	High	High	[15,34,36,38,40]

**Table 2:** Summary of the main virulence factors of the principle periodontal pathogens.

Bacteria	Component	Function	Citation
*Porphyromonas gingivalis*	LPS	Induces inflammation and activates the host immune system	[20,21]
	Gingipains	Accounts for 85% of the total proteolytic activity; In addition to increasing nutrient availability, it has an immunoregulatory purpose and plays a role in bacterial coaggregation	[19,20]
	Fimbriae	Promotes adhesion, invasion of host cells, biofilm formation, and bacterial motility	[32]
	GroEL	Induces inflammation and activates the host immune system	[97]
	Hemagglutinins	Promotes adherence to host cells and is used for heme acquisition	[98]
	Capsule	Polysaccharide layer that protects the bacterium from phagocytosis by host immune cells	[33]
*Tannerella* *forsythia*	LPS	Induces inflammation and activates the host immune system	[99]
	PrtH	Protease that plays a role in cell detachment	[41,43]
	BspA	Necessary for attachment and invasion of epithelial cells	[41,45]
	Karilysin	Immunoregulatory, causes bacterial serum resistance	[50]
	Methylglyoxal	Causes inflammation and tissue damage	[47]
	GroEL	Induces inflammation and activates the host immune system	[48]
	S-layer	Involved in coaggregation, immunoevasion, adhesion, and invasion of cells	[52,53]
*Treponema denticola*	LOS	Impacts adherence to host cells and bacterial coaggregation	[63]
	MSP	induces inflammatory responses and forms cytotoxic pores	[60,61,62]
	Dentilsin	Stimulates tissue destruction and activates C3 complement pathway; it also has an immunoregulatory purpose and plays a role in bacterial coaggregation	[64,65,66,71,72]
	Leucine-rich-repeat A	Impacts adherence and invasion of host cells, while also being important for bacterial coaggregation	[100]
*Fusobacterium nucleatum*	LPS	Induces inflammation and activates the host immune system	[79,80]
	Main Adhesin (FadA)	Required for the invasion and adhesion to host cells	[80]
	Other Adhesins (Fap2, RadD, and aid1)	Impacts binding to a variety of different bacteria and host proteins	[74,80]
	Fusolisin	Impacts nutrient availability while also having an immunoregulatory role	[81]
*Aggregatibacter actinomycetemcomitans*	LPS	Induces inflammation and activates the host immune system	[86,87]
	Leukotoxins	Disrupts immune function	[86,91]
	CTD	Induces DNA damage and cell cycle arrest which leads and apoptosis, with a pronounced impact on immune cells	[92,93]
	Fimbriae	Impacts adherence and bacterial coaggregation	[101]

**Table 3:** Summary of the longitudinal studies that attempted to correlate periodontitis with neurodegenerative disorders.

Disease Investigated	Study Design/Duration	Question	Adjusting for:	Number of Participants/Age [Years]	Results	Author, Date, Journal, and Citation
Mild cognitive impairment (MCI)	Prospective cohort study (8 years)	Does periodontitis correlate with cognitive decline?	Age, gender, race, education, income, smoking, alcohol consumption, and diabetes	N = 558 (total) Age group = 52–75 Age mean = 64.7 ± 4.3	No significant correlation was found between tooth loss at baseline and cognitive capabilities.	Supawadee Naorungroj et al. (2014) Community Dentistry and Oral Epidemiology’s [145]
Mild memory impairment (MMI)	Prospective cohort study (5 years)	Does periodontitis correlate with a decline in memory?	Age, gender, MMSE-total, education length, smoking, drinking, blood pressure, cancer, myocardial infarction, cerebrovascular disease, diabetes mellitus, hypertension, and dyslipidemia	N = 2335 (total) Age group ≥ 65 Age median = 71	Each tooth lost at baseline slightly increased the odds of developing memory impairment: (OR = 1.02; 95% Cl 1.00–1.03; *p* = 0.039)	Nozomi Okamoto et al. (2015) Journal of Alzheimer’s Disease [146]
Mild cognitive impairment (MCI)	Prospective cohort study (6 years)	Does periodontitis correlate with cognitive decline?	Age, gender, living alone, education, alcohol consumption, BMI ischemic heart disease, traumatic brain injury, and depression	N = 715 (total) N = 214 (with periodontitis) Age group = 60–96 Age mean = NA	Bone loss caused by periodontitis may be associated with faster cognitive decline: (OR = 2.2; 95% CI 1.2–3.8; NA)	Helena Nilsson et al. (2018) Journal of Clinical Periodontology [147]
Mild cognitive impairment (MCI)	Prospective cohort study (4 years)	Does periodontitis correlate with cognitive decline?	Age, gender, hypertension, diabetes, cerebrovascular/cardiovascular disease, hypercholesterolemia, depressive symptoms, body mass index, smoking status, drinking status, duration of education, and baseline MMSE score	N = 140 (total) Age group ≥ 65 Age mean = 70.9 ± 4.3	Tooth loss may be associated with cognitive impairment: (OR = 3.31; 95% Cl 1.07–10.2; *p* = 0.037)	Sho Saito et al. (2018) BMC Oral Health [148]
Mild cognitive impairment (MCI)	Longitudinal cohort study (5 years)	Is there an association between mild cognitive impairment and periodontitis?	Age, sex, smoking status, educational level, physical activity level, obesity, depression, and diabetes	N = 179 (total) Age group ≥ 75 Age mean = 80.1 ± 4.4	Severe periodontitis was significantly associated with MCI: (OR = 3.58; 95% Cl 1.45–8.87; *p* < 0.01)	Masanori Iwasaki et al. (2019) Journal of Periodontal Research [149]
Mild cognitive impairment (MCI)	Longitudinal study (median following of 5.93 years)	Is there an association between mild cognitive impairment and severity of tooth loss?	Age, gender, ethnicity, residence, marriage status, occupation, education, smoking and drinking, activities of daily life (ADL) score, physical performance score, food diversity score, social activity score, and chronic disease score	N = 11862 (total) N = 3966 (developed MCI) Age group ≥ 65 Age mean = 81.41	Higher tooth loss rate was associated with an increased risk of MCI in elderly subjects. 1–2 tooth loss per year [mild] (OR = 1.16; 95% Cl 1.04–1.29; NA) >2 tooth loss per year [severe] (OR = 1.28; 95% Cl 1.17–1.40; NA)	Shuyu Xu et al. (2021) Aging [150]
Dementia	Prospective study (longitudinal checkup every 3 years for up to 32 years)	Does severity of tooth loss correlate with dementia progression?	Age, years of education, smoking, BMI, medication use, intake of substances, coronary heart disease, stroke, hypertension, cardiovascular disease, cancer, and diabetes	N = 597 (total) Age group = 28–70 Age mean = 48 ± 8	Each tooth lost per decade since the baseline examination increased the risks of lowering the Mini-Mental State Examination score: (HR = 1.12, 95% CI 1.05–1.18; *p* < 0.05)	Kaye et al. (2010) Journal of the American Geriatrics Society [151]
Dementia	Longitudinal cohort study (5 years)	Are periodontal conditions associated with dementia onset?	Age, gender, educational level, living condition, tobacco intake, alcohol consumption, BMI, stroke, angina pectoris, diabetes, hypertension, depression, and myocardial infarction	N = 348 (total) N = 246(with some form of periodontal condition) Age group = 66–80 Age median = 70	Periodontal condition was not associated with an increased risk of dementia: (HR = 1.13; 95% CI 0.60–2.12; NA)	E. Arrivé et al. (2011) Community Dentistry and Oral Epidemiology’s [152]
Dementia	Retrospective matched-cohort study (10 years)	Is there an Association between Chronic Periodontitis and Gingivitis with Dementia?	Age, sex, monthly income, urbanization level, geographic region, hypertension, diabetes, hyperlipidemia, obesity, depression, chronic kidney disease, and stroke	N = 8828 (total) N = 2207 (with periodontitis or gingivitis) Age group ≥ 20 Age mean = NA	Patients with chronic periodontitis and gingivitis have a higher risk of developing dementia: (HR = 2.54; 95% CI 1.297–3.352; *p* = 0.002).	Nian-Sheng Tzeng et al. (2016) Journal of Neuroepidemiology [153]
Dementia	Prospective matched-cohort study (13 years)	Is there an association of chronic periodontitis with dementia onset?	Age, gender, geographic region, urbanization level, hypertension, diabetes mellitus, cardiovascular disease, congestive heart failure, atrial fibrillation, stroke, and chronic renal disease	N = 6056 (total) N = 3028 (with periodontitis) Age group ≥ 65 Age mean = 72.4 ± NA	Patients with periodontitis were at higher risk of developing dementia: (HR = 1.16; 95% Cl 1.01–1.32; *p* = 0.03)	Yao-Tung Lee et. al (2016) Journal of the American Geriatrics Society [154]
Dementia	Retrospective cohort study (10 years)	Does treatment of periodontitis affect dementia onset and progression?	Age, gender, socioeconomic status, residential urbanicity, hypertension, diabetes, and hyperlipidemia	N = 182,747 (total—all with periodontitis) N = 19,674 (did not receive treatment for periodontitis) N = 6133 (developed dementia) Age group ≥ 45 Age mean = NA	Subjects who had not received Periodontitis treatment were at greater risk of developing dementia: (HR = 1.14; 95% CI 1.04–1.24; *p* ≤ 0.001)	Ya-Ling Lee et al. (2017) Journal of the American Geriatrics Society [155]
Dementia	Prospective cohort study (5 years)	Does severity of tooth loss correlate with dementia onset?	Age, gender, occupation, educational level, tobacco intake, alcohol consumption, BMI, stroke, angina pectoris, diabetes, hypertension, and tooth brushing frequency	N = 1566 (total) N = 180 (developed dementia) Age group ≥ 60 Age mean = NA	Tooth loss may be associated with a higher risk of dementia: 10–19 teeth: (HR = 1.62, 95% CI = 1.06–2.46), 1–9 teeth (HR = 1.81, 95% CI = 1.11–2.94), 0 teeth (HR = 1.63, 95% CI = 0.95–2.80)	Kenji Takeuchi et al. (2017) Journal of the American Geriatrics Society [156]
Dementia	Retrospective matched-cohort study (10 years)	Is there an association of chronic periodontitis with dementia onset?	Age, sex, household income, smoking status, alcohol consumption, physical activity, BMI, blood pressure, fasting serum glucose, total cholesterol, and Charlson Comorbidity Index	N = 262,349 (total) N = 46,344 (with periodontitis) Age group ≥ 50 Age mean = 60.2 ± 7.3	Chronic periodontitis may be associated with a higher risk of developing dementia: Overall dementia (HR = 1.06; 95% CI 1.01–1.11; *p* = 0.015) Alzheimer’s (HR = 1.05; 95% CI 1.00–1.11; *p* = 0.042). The risk-increasing effect of chronic periodontitis on dementia tended to be stronger among participants with healthy lifestyle behaviors.	Seulggie Choi et al. (2019) Journal of the American Geriatrics Society [157]
Dementia	Retrospective matched-cohort study (14 years)	Is severe periodontitis with tooth loss a risk factor for the onset of dementia?	Age, sex, household income, insurance status, BMI, total cholesterol level, smoking status, drinking status, physical activity, hypertension, and diabetes mellitus	N = 20,230 (total) N = 10,115 (with periodontitis) Age group = 40–80 Age mean = NA	Patients with severe periodontitis with 1–9 remaining teeth were at higher risk for developing dementia: Alzheimer’s (HR = 1.08; 95% CI 1.01–1.14; *p* = 0.022) Vascular dementia (HR = 1.24; 95% CI 1.16–1.32; *p* < 0.001) Mixed dementia (HR = 1.16; 95% CI 1.09–1.24; *p* < 0.001)	Do-Hyung Kim et al. (2020) Journal of periodontal and implant science [158]
Dementia	Retrospective matched-cohort study (14 years)	Is there an association of chronic periodontitis with dementia onset?	Age, gender, influenza vaccination, income, hypertension, mental disorders, diabetes, ischemic heart disease, stroke, hyperlipidemia, chronic obstructive pulmonary disease, heart failure, liver cirrhosis, and traumatic brain injury	N = 102,036 (total) N = 56,018 (with periodontitis) Age group ≥ 50 Age mean = NA	Patients with periodontitis were at higher risk of developing dementia: (HR = 1.73; 95% CI 1.61–1.86; *p* < 0.0001) Periodontitis was associated with a higher risk of developing dementia in people with no underlying medical conditions: =0 (HR 6.16; 95% CI 5.13–7.40; *p* < 0.05) =1 (HR 1.27; 95% CI 1.10–1.46; *p* < 0.05) ≥2 (HR 1.20; 95% CI 1.09–1.31; *p* < 0.05)	Chia-Yen Lee et al. (2020) Journal of Clinical Periodontology [159]
Dementia	Retrospective longitudinal matched-cohort study (13 years with median following of 6.6 years)	Is there a difference in dementia onset between groups of people severe or mild chronic periodontitis?	Age, gender, income level, smoking status, alcohol consumption, regular exercise, BMI, systolic blood pressure, diastolic blood pressure, fasting blood glucose levels, total cholesterol levels, hypertension, diabetes, dyslipidemia, heart disease, cerebrovascular disease, depression, and Charlson Comorbidity Index	N = 8624 (with mild chronic periodontitis) N = 8624 (with severe chronic periodontitis) Age group ≥ 60 Age mean = 70.9 ± 4.8	Dementia onset was significantly associated with the severity of periodontitis. (HR = 1.15; 95% CI; 1.04–1.27; *p* = 0.01)	Seon-Rye Kim et al. (2022) Epidemiology and health [160]
Dementia	Cohort study (Follow up 7.6 ± 1.1 years)	Is there an association of deep probing pocket depths and tooth count with dementia onset?	Age, gender, civil status, disposable income, education, geographical area, and Charlson Comorbidity Index	N = 37,174 (total) N = 7992 (with ≥4 teeth and/or dental implants with probing pocket depth ≥6 mm) Age group = 40–80 Age mean = 61	Tooth loss and deep probing pocket depth does not increase the risk of developing dementia: (HR = 1.13; 95% CI 0.39–3.24; *p* > 0.05)	Jacob Holmer et al. (2022) Journal of Clinical Periodontology [161]
Dementia	Prospective cohort study (6 years)	Does tooth loss increase the risk of dementia onset?	Age, gender, marital status, denture use, education level, income level, social network, smoking status, alcohol consumption, diabetes treatment, and hypertension	N = 35,744 (total) N = 22,164 (<20 teeth) Age group ≥ 65 Age mean = 73.2 ± 5.5	Number of teeth may increase the risk of dementia onset: (HR = 1.14; 95% CI 1.01–1.28 *p* = 0.041) When the nutritional and social mediators (weight loss, vegetable and fruit intake, and regular exercise) were included, the effect was reduced to: HR = 1.10 95% CI; 0.98–1.25; *p* = 0.113)	S Kiuchi et al. (2022) Journal of Dental Research [162]
Alzheimer’s disease (AD)	Case-control study with a follow-up over a six-month period	Does periodontitis affect the speed of cognitive decline?	Age, gender, and baseline cognitive status	N = 52 (total) N = 20 (with periodontitis) Age group = NA Age mean = 77.7 ± 8.6	The presence of periodontitis at baseline was not related to baseline cognitive state: (MD = 1.1; 95% Cl -5.6–7.7; *p* = 0.8) Rate of cognitive decline: (MD = 4.9; 95% Cl 1.2–8.6; *p* = 0.01)	Mark Ide et al. (2016) PlOS One [163]
Alzheimer’s disease (AD)	Retrospective matched-cohort study (8 years)	Is there an association between chronic periodontitis and the risk of Alzheimer’s disease?	Age, gender, index year, urbanization level, hypertension, hyperlipidemia, chronic kidney disease, depression, stroke, diabetes mellitus, and traumatic brain injury	N = 27,963 (total) N = 9291 (with periodontitis) Age group ≥ 50 Age mean = 54.1 ± 10.5	The association between chronic periodontitis and AD was significant in patients that have had over 10 years of exposure to chronic periodontitis: (HR = 1.707; 95% CI 1.152–2.528; *p* = 0.0077).	Chang-Kai Chen et al. (2017) Alzheimer’s Research and Therapy [164]
Parkinson’s disease (PD)	Retrospective matched-cohort study (5 years)	Does chronic periodontitis increase the risk of PD incidence?	Age, gender, urbanization level of residence, index year, stroke, and depression	N = 320,106 (total N = 53,351 (with periodontitis) Age group ≥ 40 Age mean = NA	Chronic periodontitis may increase the risk of Parkinson’s disease: (HR = 1.43; 95% 1.32–1.55; NA)	Tsai-Ching Liu et al. (2013) Movement Disorders [165]
Parkinson’s disease (PD)	Retrospective matched-cohort study (8 years)	Does periodontitis and its severity correlate with PD incidence?	Age, gender, CCI score, urbanization level, stroke, depression, hyperlipidemia, cancer, chronic kidney disease, and traumatic brain injury	N = 16,188 (total) N = 5396 (with periodontitis) Age group ≥ 40 Age mean = 54.1 ± 10.5	Periodontitis correlates with the incidence rate of PD: (HR = 1.431; 95% CI 1.141–1.794; *p* = 0.002)	Chang-Kai Chen et al. (2017) Peer.J [166]
Parkinson’s disease (PD)	Longitudinal cohort study (10.4 years)	Does chronic periodontitis increase the risk of PD incidence?	Age, gender, income level, body mass index, lifestyle habits, such as alcohol intake, smoking status, physical activity, blood pressure, renal disease, hypertension, diabetes mellitus, and dyslipidemia	N = 153,165 N ≈ 30,480 (with periodontitis) Age group ≥ 40 Age mean = 52.8	Chronic periodontitis did not significantly increase the risk of PD incidence: (HR = 0.91; 95% Cl 0.78–1.06; *p* = 0.232) Tooth loss (≥15) correlated to higher PD incidence: (HR = 1.38; 1.03–1.85; *p* = 0.029)	Ho Geol Woo et al. (2020) Parkinson’s Disease [167]
Parkinson’s disease (PD)	Retrospective cohort study in South Korea (8 years)	Do periodontitis and its severity correlate with PD incidence?	Age, sex, smoking status, drinking habits, exercise habits, income level, BMI, diabetes mellitus, hypertension, dyslipidemia, stroke, and depression	N = 6,856,180(total) N = 903,063 (with periodontitis) Age group ≥ 40 Age mean = 55.47 ± 9.97	The severity of periodontitis correlates with the incidence rate of PD: (HR = 1.114; 95% CI 1.062–1.146; *p* < 0.001)	Eunkyung Jeong et al. (2021) Scientific report—Nature [168]

**Figure A1:**
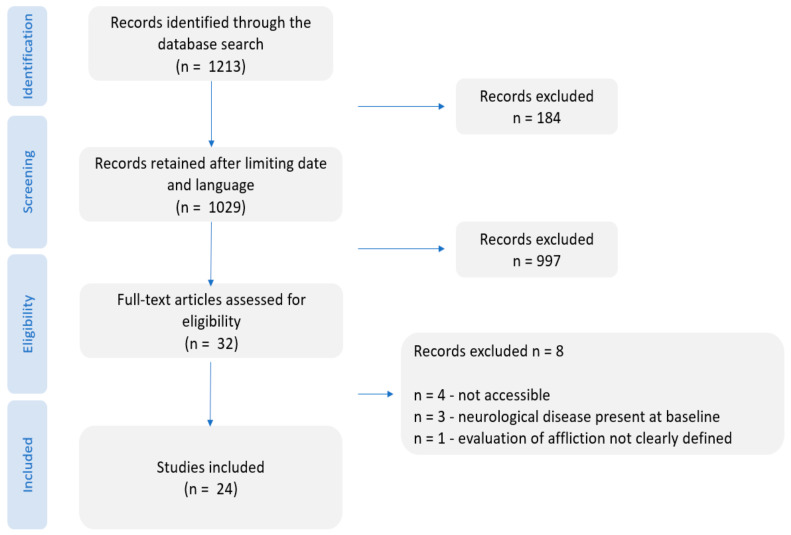
PRISMA flow diagram indicating the steps followed during the scientific literature review process of this work.

## Data Availability

No new data were created or analyzed in this study. Data sharing is not applicable to this article.

## Appendix A

### Appendix A.1. Systemic Extraction of Data

The only section that required a specific approach was Table 3. Data curation was performed in alignment to PRISMA guidelines.

This was conducted by two authors using the PubMed advanced filter with the search terms: periodontitis; periodontal disease; dementia; motor neuron disease; amyotrophic lateral sclerosis; multiple sclerosis; Parkinson; Parkinson disease; Alzheimer disease; neurodegenerative disease; neurodegenerative. These search terms were then mixed using Boolean operators (AND, OR, and NOT) and MeSH terms. The results of the search are shown below Figure A1.

### Appendix A.2. Eligibility Criteria

We focused on studies written in English that were published between 1993 and 5 May 2023. The inclusion criteria were as follows: 1—human studies; 2—the analysis of the link between periodontal disease and neurodegeneration was one of the objectives of the study; 3—Evaluation methods for periodontal disease was clearly defined; 3—Evaluation methods for neurodegenerative disease was clearly defined; 5—studies observed the longitudinal relationship between periodontal disease and neurodegeneration; 6—subjects were not afflicted by the observed disease when examined at baseline.
